# Step-and-Repeat Nanoimprint-, Photo- and Laser Lithography from One Customised CNC Machine

**DOI:** 10.1186/s11671-016-1341-9

**Published:** 2016-03-08

**Authors:** Andrew IM Greer, Benoit Della-Rosa, Ali Z. Khokhar, Nikolaj Gadegaard

**Affiliations:** School of Engineering, University of Glasgow, G12 8LT Glasgow, UK

**Keywords:** Flash, Imprint, Replication, Stepping, Resist, Nanolithography

## Abstract

The conversion of a computer numerical control machine into a nanoimprint step-and-repeat tool with additional laser- and photolithography capacity is documented here. All three processes, each demonstrated on a variety of photoresists, are performed successfully and analysed so as to enable the reader to relate their known lithography process(es) to the findings. Using the converted tool, 1 cm^2^ of nanopattern may be exposed in 6 s, over 3300 times faster than the electron beam equivalent. Nanoimprint tools are commercially available, but these can cost around 1000 times more than this customised computer numerical control (CNC) machine. The converted equipment facilitates rapid production and large area micro- and nanoscale research on small grants, ultimately enabling faster and more diverse growth in this field of science. In comparison to commercial tools, this converted CNC also boasts capacity to handle larger substrates, temperature control and active force control, up to ten times more curing dose and compactness. Actual devices are fabricated using the machine including an expanded nanotopographic array and microfluidic PDMS Y-channel mixers.

**Electronic supplementary material:**

The online version of this article (doi:10.1186/s11671-016-1341-9) contains supplementary material, which is available to authorized users.

## Background

Nanoimprint lithography (NIL) has been well recognised as a mechanism capable of facilitating the accurate replication of etched electron beam lithography (EBL) patterns at high speed, by transferring millions of nanofeatures in unison [1–8]. Nanoimprint boasts financial benefits as the set-up, maintenance and running costs involved are a fraction of the EBL equivalent. The technology can be split in two directions: wafer-scale imprints to replicate a single complex design [1, 9] and step-and-repeat processing whereby a smaller stamp is tiled to replicate devices and scale processing to the wafer level [10–12]. Despite the increase in speed for large area patterning in contrast to EBL, a commercial nanoimprint step-and-repeat machines may costs in the region of US$10 million [13, 14]. However, a commercial computer numerical control machine (CNC machine) may be purchased for less than US$650. Here we document the successful conversion of a CNC machine into a multifunctional lithography tool, capable of step-and-repeat nanoimprint lithography with UV-sensitive resists. In this work, the versatility of the machine is explored, a series of resists are examined in the machine and the capacity to perform both photo- and laser lithography is also demonstrated.

Laser lithography using a CNC machine offers flexible freeform style of exposure using the micrometre scale precision and inherent curve tracing functions of the CNC controller. Such tool versatility is not available on conventional photolithography tools. The reason photolithography upon a step-and-repeat machine is interesting is primarily because a small mask may move the entire length of a 6-in. wafer (or even further). The majority of mask aligning lithography tools do not facilitate more than around 7 mm of displacement. This reduces the size of the required mask which is desirable if EBL is required to produce the initial photomask. In addition, greater pattern versatility is facilitated at the lithography stage.

**Fig 1:**
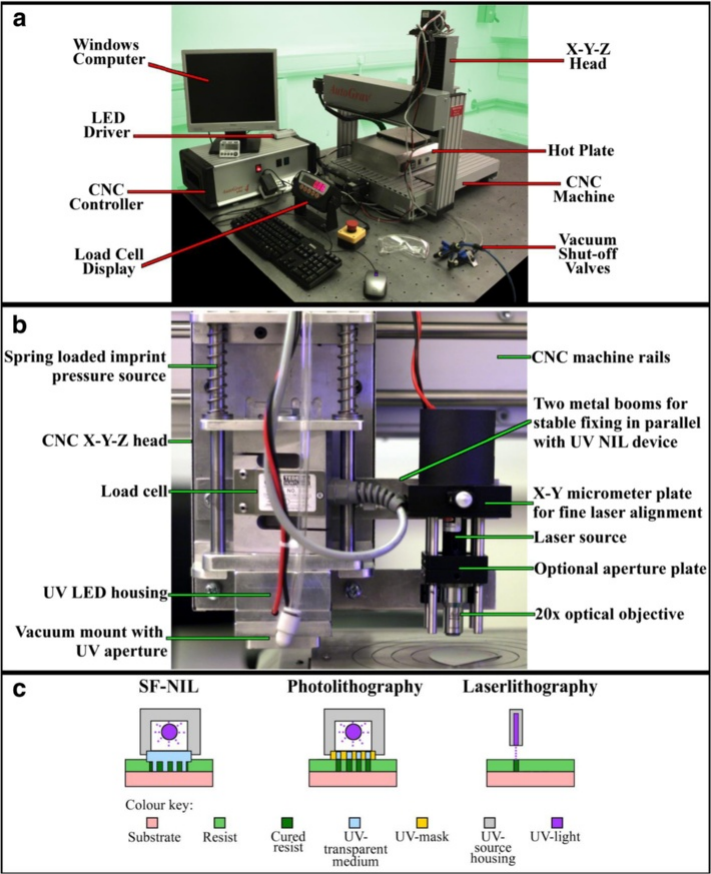
**a** Annotated photograph of the converted CNC machine. **b** Annotated photograph of the custom-built lithography tool attachment for the CNC X-Y-Z head. **c** Schematic representation of the three discrete lithographic disciplines available on the converted CNC machine

Before the performance of the multifunctional lithography tool may be evaluated, the tool components and assembly need to be introduced. A commercial CNC machine (SMC OS Microstep USB controller, AG2SO Spindle Drive and cncGraf Pro software for Windows) was purchased from Auto Grav. The machine inherently possesses the ability to move an X-Y-Z head in relation to a base plate for normal CNC processing. The tool is also equipped with a 240 V AC switchable spindle output to control an additional peripheral (commonly a router) which in this case is used to activate a UV LED. A mount and imprinter head were custom furnished to attach onto the CNC X-Y-Z head. Annotated photographs of the customised CNC machine are displayed in Fig. 1. The imprinter head consists of a downward-facing vacuum mount with a bored centre which holds a 365-nm UV LED (LZ4-00U600 from LED Engin). This section is suspended below sliding rules on twin vertical stanchions. The stanchions are secured to a mounting bracket on the CNC X-Y-Z head. Surrounding the guide rails are springs. These springs are responsible for applying the actual imprint force and therefore may be changed if a higher or lower load is required. In order to monitor the load being applied by the imprinter, an S-type load cell from Tedea-Huntleigh (model 614) is positioned between the springs and the imprinter head. The output from the load cell is sent to a Revere Transducers VT100 seven-segment display so that a user may observe how much load is being applied in real time as Z position is changed. The CNC controller also features a five-pin DIN connector with two of the pins conditioned to activate relays, one of these pins was utilised to control a constant current driver for a 405-nm wavelength, 1-mW laser sourced from Roithner LaserTechnik. This laser was mounted inside a laser cage which was also fixed to the X-Y-Z head. The laser cage mount assembly is shown in the annotated photograph of Fig. 1b. There is an X-Y micrometre pinned to the laser source for performing fine adjustments to the laser position within the cage. Below the laser, a pin-hole aperture may be mounted to ensure beam uniformity and circularity. At the bottom of the laser cage is a ×20 optical objective with numerical aperture of 0.32 which reduces the spot size and increases the intensity of the beam. In order to facilitate embossing at elevated temperatures, a hot plate was sourced from Electronic Micro Systems Ltd. This particular model of hot plate (1000-1 Precision Hot Plate) has a robust full metal construction and a vacuum port designed to hold samples up to 150 mm in width, making it suitable as a wafer mounting base for this stepping machine. All of the high performance equipment documented above was purchased for less than US$11,000.

As described earlier, this custom-built machine has three modes of operation: NIL, photolithography and laser lithography (Fig. 1c depicts a schematic of the lithography mechanisms). To operate the tool, a substrate (of up to 150 mm square) must be pre-coated in UV-sensitive resist before mounting on the vacuum port of the hotplate base. The feet of the hotplate may be lowered or raised to level the system. To perform photolithography or NIL, a photomask or UV-transparent stamp of base 25 mm square needs to be loaded into the recessed vacuum port on the imprinter head. The CNC software (cncGraf Pro) may then be used to position the stepper head and lower it into contact with the resist coated substrate. As the mask/stamp contacts the substrate, the springs on the stepper head compress and the compression force is measured by the load cell. The CNC software can then be used to turn on the UV LED until the resist below the mask/stamp is cured. A calibration substrate is initially required to determine both the load and exposure dose required for optimum pattern transfer because the curing time is dependent on the thickness and type of resist used and the forward current applied to the UV LED as well as composition and thickness of the mask/stamp. Once the desired load and exposure dose are determined, the process of stepping out a pattern may be automated with G-code programming (the standard CNC language) or discrete moves may be initiated by the user as and when required. Provided the substrates are flat, the calibration load may be correlated to a Z position and utilised in the automation code as the imprint position (because one cannot assign a desired pressure in G-code but one can assign a Z position). The LED may be turned on with the G-code by triggering the spindle output. The automation code also requires a Z position above the substrate in order to define a safe level of withdrawal so that any desired change to the X and Y position may be accommodated. Once the desired series of exposures/imprints have been obtained, the substrate may be removed from the machine as the lithography stage is now complete. The third lithography mechanism is laser curing. The laser source is only designed to cure a point location on a resist-coated sample surface but through automated movement may expose the resist through freehand style. The speed of motion and intensity of light may be varied. The laser assembly is set up so that it is positioned at a distance defined by the objective. With the ×20 objective present in this report, the working distance is 15 mm.

## Methods

### Tool Capacity

The native working conditions were first evaluated without the presence of any stamps, masks or samples. The head speed, minimum imprint pressure, base plate temperature and controllable dose range for each light source were measured. To determine the minimum load which the imprinter head was capable of applying, a readout was taken from the inbuilt load cell. The irradiance was measured with a Thorlabs PM100 USB power metre equipped with a S302C thermal power sensor and IR filter in continuous wave mode calibrated for 365 nm. The forward current across the sources was varied from 250 to 700 mA using variable current power supplies, and the radiated power was recorded below each head assembly. The impedance of different mask/stamp mediums upon the transmitted power was also measured over the same range of drive current.

### Nanoimprint Lithography Analysis

UV-NIL is the lithographic method, offered by the custom-built machine, which offers the highest single exposure resolution. By first defining and etching a mould using EBL and reactive ion etching (RIE), one may cast a UV-transparent elastomer such as PDMS onto the mould, relieve it and mount it on the stepper head (with the assistance of a quartz or glass back plate) to create a UV-NIL stamp (the details of how to fabricate such a device are detailed in Section S2 of Additional file 1). This style of stamp is advised because its elastomeric qualities act as a self-levelling system. Step-and-flash nanoimprint lithography (SF-NIL) may then be carried out using the machine. G-code can be compiled to automatically step out a full wafer of pattern for rapid replication or scaling of the original stamp pattern (an example of which may be found in the Additional file 1).

Two UV-NIL resists were acquired to test the capacity of the custom-built system to operate as a step-and-repeat machine. The two resists were the commercially available AMO NIL MMS4 and a novel resist under development by Delo Industrial Adhesives presently known as DELO-KATIOBOND OM VE 110707 (referred to here after as Delo-Katiobond). Both resists were selected because they may be cured with 365-nm wavelength radiation in an air atmosphere. However, unlike AMO NIL MMS4, Delo-Katiobond has a purely organic chemistry so it may be etched in an oxygen plasma, thus enabling non-stringent residual layer removal [15].

AMO NIL MMS4 was used neat, and Delo-Katiobond was diluted with 7.25 parts chlorobenzene. Both resists were pre-spun at 5 krpm for 1 min onto 500-μm thick Si and effectively patterned using the custom-built step-and-repeat machine fitted with a 3-mm thick PDMS stamp featuring a 1-mm thick quartz backing. The imprint pressure was determined via a load cell on the imprinter tool which monitors the force during operation. The curing dose was determined by exposing resist for different durations and correlating it to the output power detected under the stamp. The maximum number of iterations per soft-PDMS stamp and radiation bleed was determined by scanning electron microscopy (SEM) inspection of the imprinted nanofeatures. When the examination by SEM indicated that the texture had deviated from discrete circular nanopillars, the stamp was considered soiled and no longer effective as a stamp. Using the calibrated scale of the SEM tool, the inter-pattern distance between two consecutive overlapping imprints was measured.

To illustrate the operation of SF-NIL, a topographical array with unit side 7 mm was stepped out on Si using Delo-Katiobond. The array which comprised various sub-sections was spread across a 4-in. wafer. The entire wafer was etched uniformly to a depth of 65 nm (the RIE parameters are included in Additional file 1 Section S3). The nanopattern within each unit of the array consists of disordered 200-nm diameter pillars which are presently of imperative importance to stem cell research and can only be fabricated via EBL [16–18]. The EBL process is highly time-consuming for this particular design at a rate of five and a half hours per square centimetre. Rapid array production via replication of the EBL pattern through SF-NIL is thus not only a good demonstration of the technology but useful for furthering stem cell studies.

### Photolithography Analysis

Once the intrinsic machine parameters were determined, the functionality of the tool was examined. The capability of the machine to work as a photolithography tool with several commercially available resists which are frequently utilised by microfabricators was explored. The resists examined were Microposit S1800 G2 series positive tone resist, Clariant AZ 4500 series positive tone resist and MicroChem Nano SU-8 negative tone resist. As stated in the introduction, the motivation for performing photolithography with a step-and-repeat tool is that it reduces the area of any EBL-defined photomask and facilitates greater pattern flexibility during the lithography stage. The photomask deployed was composed from a 25 × 25 × 1 mm piece of quartz with an aluminium face featuring 35 7-mm long by 5-μm wide UV-transparent lines at 200-μm pitch defined by EBL (the details of how to fabricate such a device are detailed in Additional file 1 Section S1). For photolithography, the head was brought into hard-contact (at minimum load) and the exposure dose (forward current across the UV LED and exposure duration) was varied for each tested resist to determine the optimum curing conditions. In all of the aforementioned tests, the base plate was kept at room temperature.

For the photolithography tests, the S1805 photoresist was spun to 500-nm thick and the AZ4562 was spun to 5-μm thick as was the Nano SU-8 resist. Post-exposure, the S1805 was chemically developed in 1:1 Microposit Developer Concentrate: RO water for 75 s and rinsed in RO water. The AZ4562 was developed in 1:5 AZ400K developer: RO water for 2 min and the Nano SU-8 is chemically amplified so it required a pre-exposure and a post-exposure bake at 95 °C for 2 min before being developed in agitated Microposit EC Solvent 11 for 2 min.

Using the described photomask, one may step out lines of varying length, width and pitch. To demonstrate this capability, the CNC head was displaced by various lengths in orthogonal directions between exposures of S1805. The developed resist was examined using optical microscopy to determine the ultimate dimensions and stitch errors of the exposed pattern.

### Laser Lithography Analysis

In the laser lithography testing, no photomask was required as designs are created by freeform control so the maximum output power was used and the head speeds were varied to detect the minimum line width possible with the set-up for Microposit S1800 G2 series positive tone resist and MicroChem Nano SU-8 negative tone resist. Again, the base plate was kept at room temperature for this work.

To showcase the capability of the laser lithography, a microfluidic mixing channel was designed and uploaded as G-code to the machine. In order to produce the channel in a functional material for microfluidics, the pattern was first exposed in negative tone SU-8 resist and developed (using the same development conditions as for the photolithography above). The microfluidic relevant material poly-dimethylsiloxane (PDMS) was then cast on to the SU-8/Si sample before being released and bonded to quartz to create a functional microfluidic mixer.

## Results and Discussion

### Tool Capacity

The intrinsic performance characteristics of the custom-built stepper are listed in Table 1. Plots of laser spot power and LED irradiance against drive current in the presence and absence of relevant filters may be found in Additional file 1 Pages S7. It is shown in Table 1 that at a forward current of 700 mA (the recommended drive current for the LED), the irradiance (or power density) of the LED employed is 102 mW/cm^2^ which is multiple times more powerful per unit area than many commercial photolithography tools. This relatively high dose is a result of the inclusion of a high power LED and the stepper head featuring a reflective enclosure with 1 × 1 cm^2^ aperture. Table 2 compares the custom-built lithography tool to the SUSS Mask Aligner MA6 and the EVG 770 NIL Stepper. The ability to modulate the irradiance level and the capacity to attain a relatively high irradiance level are notable assets of this custom-built multifunctional UV-lithography tool. Table 1 indicates the maximum output power for the light sources used, but the tool is not restricted to operating with these sources. Higher power light sources may be incorporated should the reader wish higher dose levels or faster laser writing.

**Table 1:** Performance characteristics of the custom-built multifunctional UV-lithography tool

Characteristic	Minimum	Maximum
LED irradiance (mW/cm^2^)	45.0	102.0
Laser power (mW)	0.18	1.00
Imprinting load (kg)	0.10	High (not measured)
Length displacement (mm)	0.001	390.000^a^
Breadth displacement (mm)	0.001	290.000^a^
Head speed (mm/s)	0.001	25.000
Working temperature (°C)	Room Temperature	150

^a^Although the CNC head may displace to the tabulated maximum, with the documented hotplate functioning as the substrate mount, the displacement is limited to the mount area of 150 mm square

**Table 2:** Comparison of the converted CNC machine criteria to purposely built commercial counterparts (data for which was determined from official data sheets; Suss power density was given for the discrete wavelength, whereas EVG was approximated for broadband spectrum range 300–500 nm)

Model	Custom tool	Suss MA/BA6	EVG 770 NIL Stepper
Price	$	$$$$	$$$$$
Power density (mW/cm^2^) @ 365 nm	102.0	7.1	~20.0
Desktop unit	Yes	No	No
X/Y accuracy (μm)	<50.0	0.5	0.5
Optical alignment	No	Yes	Yes
Maximum substrate (mm)	390	150	300
Active force control	Yes	No	Yes
Imprint environment	Air	Air	Vacuum/inert gas
Temperature control	Yes	No	No
Laser lithography	Yes	No	No

Although Table 1 does not provide a value for the maximum imprinting load, hundreds of kilogrammes may be applied with this CNC machine (by over loading the springs) which is in excess of the load required to smash 25 × 25 × 1 mm quartz based stamps; hence, why this parameter has not been evaluated. This parameter is not considered to be as important as minimum load for the application of S-NIL as it is unlikely one would desire high loads on a soft, deformable stamp. The CNC head may displace to 290 and 390 mm in orthogonal directions, but when the documented hotplate is used as the substrate mount, the head displacement is limited to the face area of the hotplate, 150 mm^2^.

### Nanoimprint Lithography Analysis

UV-NIL is the mechanism which facilitates the highest resolution of lithography for the custom-built machine and is considered by the authors to be the most beneficial of the three lithographic processes on offer. This is primarily due to the speed at which SF-NIL may be performed. The disordered nanospot design utilised in this work takes around five and a half hours or 19,800 s to write a square centimetre of in poly-methyl methacrylate (PMMA) using an EBL tool (Vistec VB6 UHR EWF). However, this custom-built step-and-repeat machine can define a square centimetre in just 6 s with no chemical development required. As previously discussed, different resists possess different sensitivities. The resist which cured 1 cm^2^ in 6 s was Delo-Katiobond, a novel resist presently under development. For comparison purposes, its performance is evaluated in Table 3 against a known UV-NIL resist, AMO NIL MMS4. As may be seen from Table 3, the Delo-Katiobond is more sensitive than its AMO counterpart. The stitch error between exposure steps in each resist is related to the resist sensitivity. Trivially, larger levels of radiation will induce a larger radiation bleed radius (outside of the stamp area). Thus, Delo-Katiobond suffers a smaller degree of radiation bleed than AMO NIL MMS4.

**Table 3:** Tested resist performance criteria

Performance criteria	AMO NIL MMS4	DELO-KATIOBOND OM VE 110707
Imprint pressure (kPa)	475	376
Curing dose (J/cm^2^)	8.0	0.4
Etched by O_2_ plasma	No	Yes
Si:resist etch ratio	1:1	4.87:1
Maximum iterations/soft-PDMS stamp	14	34
Radiation bleed (μm)	300	70

Although this work found that just 34 imprints into Delo-Katiobond were possible before the soft-PDMS stamp deployed started to become fouled, work published by Schmitt et al. reports that over twice as many iterations (80) are achievable using soft-PDMS and that hard-PDMS may be even more fruitful with approximately 500 imprints achievable [19]. The difference between the work presented here and the work of Schmitt et al. is that in their work, a commercial stepper was used, Nano Patterning Stepper NPS300, with this set-up, a single drop of resist may be applied prior to each imprint so radial bleed is not an issue as there is only uncured resist below the stamp area [19]. This means a far larger exposure dose may be applied. When the resist is cured for longer, it becomes more cross-linked, meaning the amount of solid content and the strength of the resist are greater so stamp contamination is lower per imprint and thus more imprints are achievable from each stamp. By modification of the custom-built tool being used for this work via the incorporation of a drop dispenser unit on the stepper head, instead of spinning resist onto the samples pre-imprinting, this issue may be resolved.

**Fig. 2:**
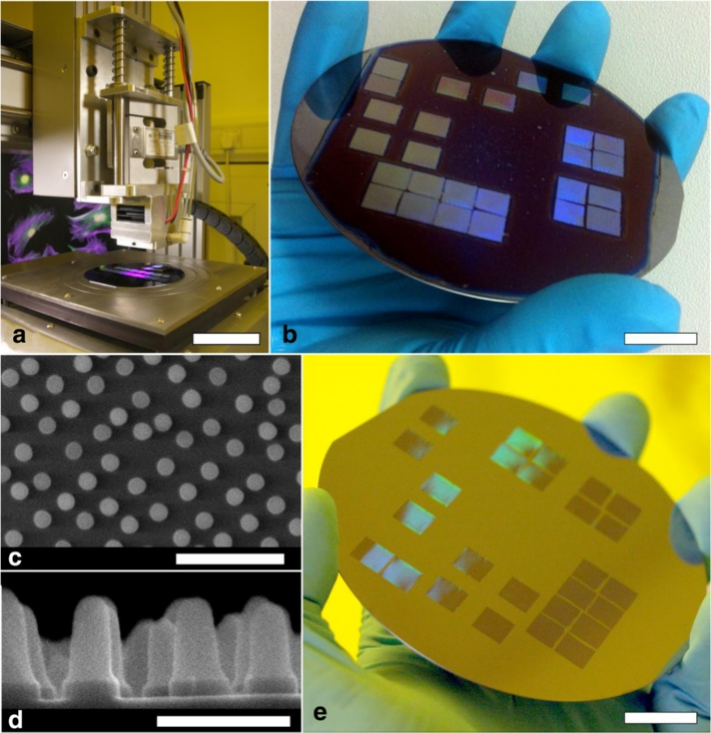
**a** Photograph of the custom-built tool being used to pattern a 4-in. Si wafer. *Scale bar* = 8 cm. **b** Photograph of a 4-in. Si wafer coated with Delo-Katiobond featuring stepped out 7 mm^2^ imprints of disordered nanopillars. *Scale bar* = 14 mm. **c** Top-down SEM image of the imprinted 200-nm diameter disordered nanopillars in Delo-Katiobond. *Scale bar* = 1 μm. **d** Cross-section at 90° tilt of the same sample as **c** following Si etching. *Scale bar* = 500 nm. **e** Photograph of the 4-in. wafer shown in **b** following Si etching and stripping of the resist in piranha etch

The fabrication of a Si nanotopographical array featuring a known osteoinductive nanofeature layout [20] is documented photographically in Fig. 2. Delo-Katiobond was used as it outperformed AMO NIL MMS4 in terms of stamp iterations, curing time, radiation bleed and ease of pattern transfer into Si. As may be observed from Fig. 2, the array of disordered nanopillars was first stepped out by the machine into Delo-Katiobond, then transferred into the Si by means of RIE and finally, the remaining resist mask was stripped in piranha etch (3:1 parts sulphuric acid: 30 % hydrogen peroxide).

### Photolithography Analysis

**Fig. 3:**
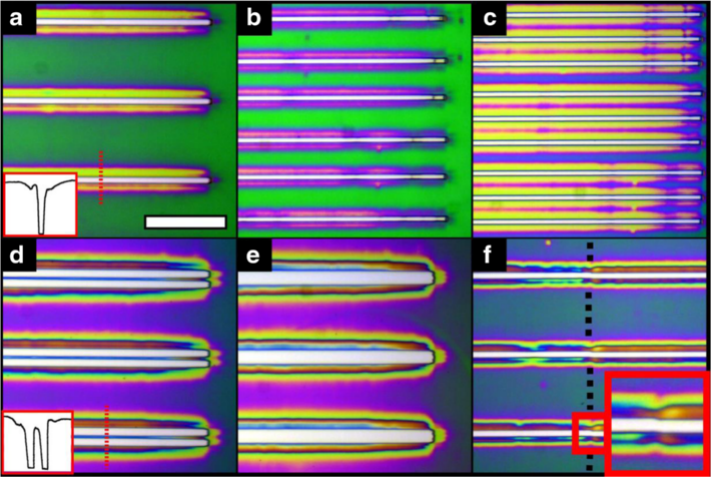
Optical micrographs of developed exposures in S1805 resist (all images are at the same magnification and the scale bar of (**a**) 200 μm): **a** a single exposure of the mask (*inset*: profile from *dashed line*), **b** a double exposure where the mask has been moved 100 μm in the transverse direction to the grating between exposures, **c** a triple exposure where the mask has been moved in 65 μm steps in the transverse direction to the grating between exposures, **d** a double exposure where the mask has been moved 30 μm in the transverse direction to the grating between exposures to create a 10 μm wide protrusion (*inset*: profile from *dashed line*), **e** a double exposure where the mask has been moved 20 μm in the transverse direction to the grating between exposures to widen the grating features and **f** a double exposure where the mask has been moved 7 mm in the direction of the grating between exposures to double the length of the features (the *dashed black line* indicates the stitch position). (*Inset*) ×2 magnified close-up of the stitch position for one line of the grating

The capacity for the custom-built machine to perform photolithography upon a variety of resists is demonstrated in Table 4. Microposit S1805, Clarient AZ 4562 and MicroChem Nano SU-8 3005 were all exposed through a photomask using the LED source. The different resists are designed for different functions and naturally possess different sensitivity to 365 nm radiation. Table 4 lists the dose which was found to be optimum to clear out each resist as well as the resist dimensions and exemplary optical micrographs of each exposure. Figure 3 displays optical micrographs of developed S1805 which demonstrates how the computer-driven, motorised UV source may be repositioned to produce various patterns in resist without manual adjustment or optical alignment. The figure shows the S1805 resist exhibiting a range of colours. The colour varies due to changes in the resist thickness. Profile analysis was carried out to identify the film thickness for the prominent colours of Fig. 3 and may be found in the Additional file 1 Section S6. The white colour is the underlying silicon. Parts (a) to (c) demonstrate how the periodicity of a grating may be doubled and tripled, respectively. Part (d) illustrates that narrow protrusions may be accurately produced through double exposure lithography. In parts (e) and (f), the exposed gratings are increased in width and length, respectively, through sequential exposures. As there is no camera incorporated on the current set-up, optical alignment is not possible. This is a concern for hierarchical lithography and the stitching of patterns (as observed in Fig. 3f). However, it is to be expected that more accurate alignment would be achievable if a camera system was incorporated. On the nanoscale, even the commercial tools bode poorly with 0.5 μm quoted as their alignment accuracy. Evidently, step-and-repeat lithography is rarely used to stitch precision nanopatterns; it is however frequently used to replicate discrete designs, the application which this converted CNC machine is primarily designed for. The machine position accuracy was found to be independent of head speed, direction, number of movements or distance moved (as may be observed from tabulated results in the Additional file 1 pages Section S4). Statistically, from 15 movements of 7 mm, it was found that the mean displacement was 25.8 μm +/− a standard deviation of 20.3 μm.

**Table 4:** Optimum machine parameters for photolithographic exposure of various popular photoresists using the UV-LED light source and a grating mask of 5 μm × 7 mm openings at 200 μm pitch, (*inset*: profile from *dashed lines*)

Resist	Microposit S1805	Clariant AZ 4562	MicroChem Nano SU-8 3005
LED current	700 mA	700 mA	700 mA
Dose	135 mJ/cm^2^	404 mJ/cm^2^	359 mJ/cm^2^
resist thickness	0.5 μm	4.5 μm	5.7 μm
Line width	13.0 μm	7.3 μm	26.0 μm
Optical micrograph			

Image scale bars = 200 μm

### Laser Lithography Analysis

**Fig. 4:**
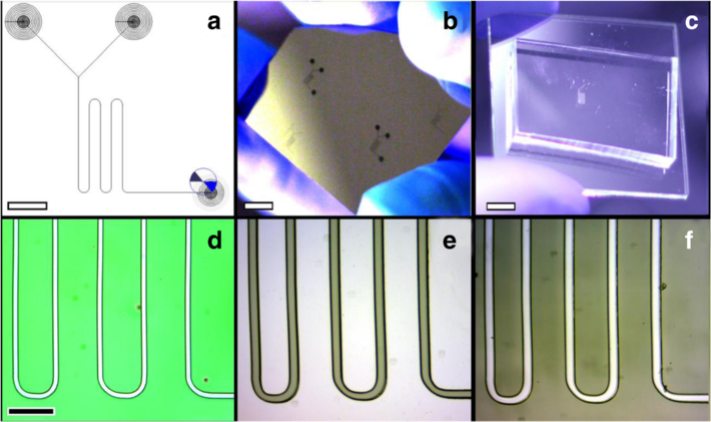
**a** Screen capture of the microchannel mixer design being exposed with the laser lithography tool. *Scale bar* = 600 μm. **b** Photograph of a developed SU-8 pattern on Si. *Scale bar* = 2.5 mm. **c** Photograph of a relieved PDMS casting from the sample shown in **b** bonded to a quartz slide to seal the hollow channel. *Scale bar* = 2.5 mm. **d**–**e** Optical micrograph showing part of the design exposed via laser lithography into **d** S1805 and **e** Nano SU-8 post development. **f** Optical micrograph of the PDMS/quartz device shown in **c. d**–**f** At the same magnification, *scale bar* shown in **d** = 150 μm

The 405-nm laser source also worked effectively to expose both tested resists, S1805 and Nano SU-8. A screen capture of the microchannel mixer design as it was being exposed by the CNC machine is shown in Fig. 4a. It was discovered that the optimum writing speed for exposing S1805 with a beam spot power of 0.21 mW (following transmission through the ×20 optical objective) was 0.3 mm/s, whereas for SU-8, the optimal writing speed was 0.1 mm/s at the same spot power. Optical micrographs of the exposed resists are shown in Fig. 4d, e. The line width obtained with these conditions were 22.7 and 47.5 μm for S1805 and SU-8, respectively. A PDMS casting of the SU-8/Si sample was relieved and bonded to quarts as shown in Fig. 4c, f to create a functional microchannel mixer./

## Conclusions

Here it has been demonstrated for the first time that a CNC machine may be successfully converted into a multifunctional UV-lithography tool capable of photo-, nanoimprint- and laser lithography. All of the lithography methods functioned properly on a variety of chemical resists. Advancements over commercial alternatives include up to 15 times more curing dose, bench-top compactness, larger substrate capacity, temperature control and the unique ability to perform freeform laser lithography. Although the presented set-up does not include any means of optical alignment, it was discovered that the machine is inherently capable of 50 μm lateral tolerance, which is suitable for nanoscale device replication but not nanopattern stitching. Expensive, commercial tools (such as the EVG 770 NIL Stepper) offer a far superior alignment tolerance of 0.5 μm, but ultimately, this is still not precise enough for nanoscale stitching. Actual devices were fabricated using the machine including a microfluidic PDMS Y-channel mixer, via the laser adaptor, and an etched nanotopographical array for cell culture experiments, using the NIL head. The cell culture profile was transferred to novel NIL resist, DELO-KATIOBOND OM VE 110707, 3300 times faster than if it had been defined by EBL in PMMA. This CNC conversion offers an affordable alternative to purposely built commercially available tools, which will be of interest to small research groups worldwide as it facilitates rapid production and high throughput micro- and nanoscale research on small grants, ultimately enabling faster and more diverse growth in this field of science.

## Additional file

Additional file 1: Increased technical details and exemplary material. (DOCX 2.02 mb)
