# Supplementary material for: Step-and-Repeat Nanoimprint-, Photo- and Laser Lithography from One Customised CNC Machine
Source: Nanoscale Res Lett. 2016 Mar 8;11:129. doi: 10.1186/s11671-016-1341-9 (PMC4783314; doi:10.1186/s11671-016-1341-9)
Supplement: Additional file 1: — Increased technical details and exemplary material. (DOCX 2.02 mb) [file 11671_2016_1341_MOESM1_ESM.docx]

Step-and-Repeat Nanoimprint-, Photo- and Laser Lithography from One Customised CNC Machine

Andrew IM Greer ^1,*^

Email andrew.greer@glasgow.ac.uk

Benoit Della-Rosa ^1^

Ali Z. Khokhar ^1^

Nikolaj Gadegaard ^1^

^1^School of Engineering, University of Glasgow, G12 8LT, Glasgow, UK

Additional file 1

S1. Photomask fabrication

A grating mask was designed with thirty-five 7 mm x 5 µm lines at 200 µm pitch using electron beam lithography into PMMA resist above a 25 x 25 x 1 mm quartz substrate with its face coated in 50 nm thick Al. The PMMA resist was developed in 1:1 MIBK:IPA solution at 23 °C for 1 minute, rinsed in IPA, blown dry and exposed to an oxygen plasma of 40 Watt for 1 minute to remove any residual resist. Then the sample was placed in Al wet etch solution (12 parts Nitric acid, 192 parts Orthophosphoric acid and 36 parts RO water) for two minutes to transfer the pattern from the PMMA into the Al layer. The PMMA resist was then cleared off using acetone leaving a photolithography hard mask suitable for mounting in the custom build stepper.

S2. UV-NIL stamp fabrication

It is essential that the stamp used with the imprinter is transparent for the wavelength needed to cure the resist. It is also beneficial to incorporate a mesa and aperture. Quartz is a suitable material for a stamp however the dry etch rate for quartz is low so producing a mesa of tens of microns in depth would require days of etching. Wet etching is possible but is more isotropic and masks are more susceptible to delaminating. A disadvantage of quartz is that the rigidity means that the stamp needs to be perfectly aligned to the substrate for conformal contact and the imprinter head needs to be nanometre level to the base plate of the machine. However it was discovered that imprints without mesas are possible providing resist is deposited by drop-dispensing. This discounts the degree of parallelism required of the stamp as the resist will flow to fill the cavity space. This is shown in Figure S1, here a 25 x 25 mm quartz nanopillar stamp with no mesa has been pressed onto a drop of UV-curable resist. The resist has spread to the edges of the quartz stamp, the nanoscale impressions are visible and there is no visible air trapping; however delaminating has occurred in one corner.


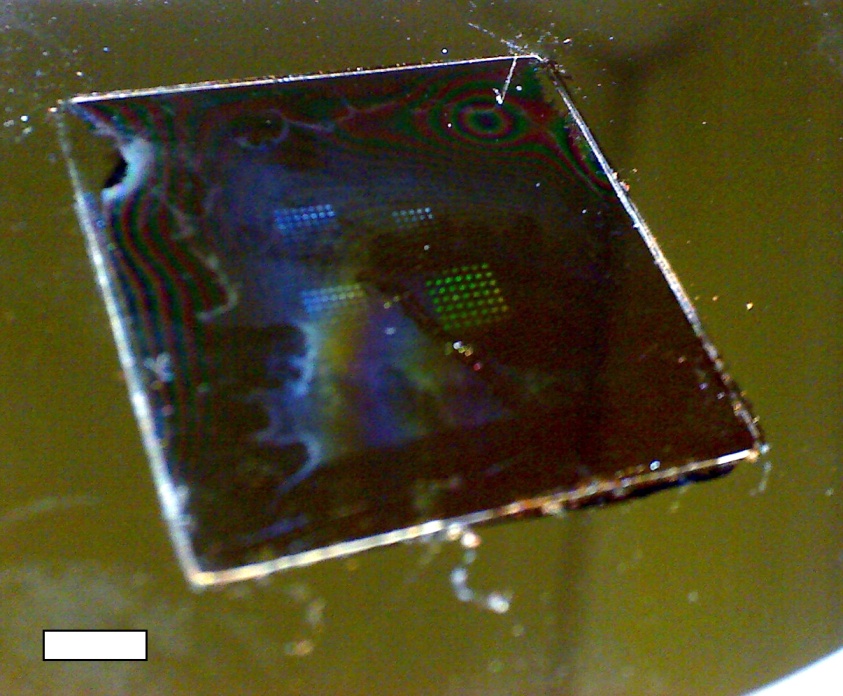


**Figure S1. By drop-dispensing UV-curable resist - hard, flat stamps may be accommodated by the customised CNC tool as the resist flows to fill the cavity below the stamp and accommodates non-level alignment. Scale bar = 5 mm.**

An alternative material which may be used for stamp fabrication is polydimethylsiloxane (PDMS). PDMS combats all of the issues associated with quartz as mesas may be produced by casting instead of etching and the flexible nature of the material allows conformal contact with more forgiving levelling. Soft PDMS is not rigid enough to mount directly on the bored out stepper vacuum-port head without deformation of the PDMS membrane so mounting upon a flat 25 mm square quartz back-plate is required.

To fabricate a nanoimprint stamp for the step-and-repeat machine, a piece of Si is first patterned and etched using conventional Si based EBL processing. This piece of Si is to become the foundation of a mould fore casting a PDMS mesa by coating it with SU-8 resist. The thickness of the resist determines how deep the mesa will be. A square photolithography mask is aligned to the nanopattern and used to define the mesa. Once the SU-8 has been developed, silane vapour treatment may be applied. This completes the mould for which at least ten iterations of PDMS may be cast. Figure S2 displays a photo of a completed mould.


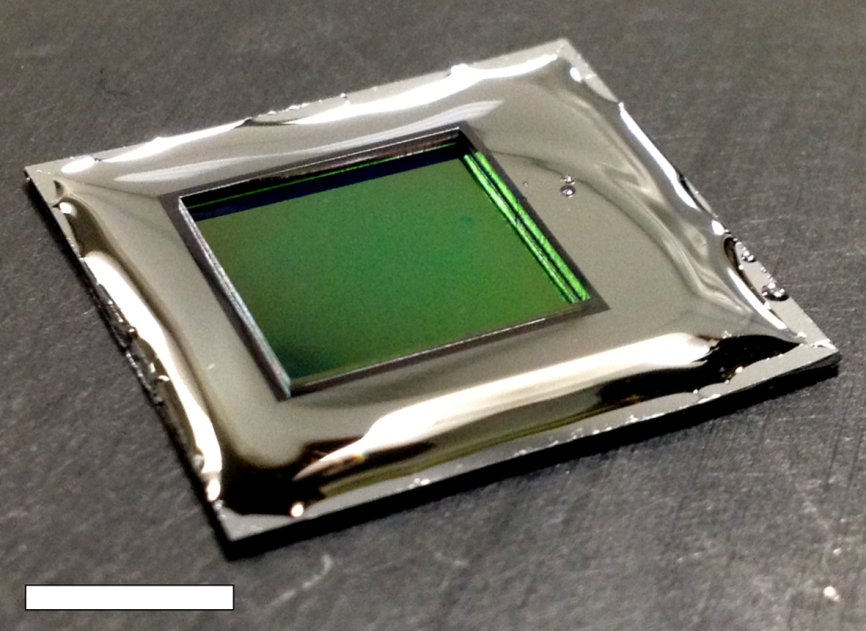


Figure S2. Exemplary mould which PDMS may be cast upon to fashion a mesa structure with nanopatterned face. 500 µm thick Si patterned with nanopillars inside a 10 mm square opening in a 420 µm thick SU-8 frame, scale bar = 8 mm.

Sygard 184 was purchased from Dow Corning and mixed in a ratio of 5:1, degassed and poured onto the Si/SU-8 mould before a further degassing and curing in an oven at 70 ^o^C overnight (>12 hours). Thereafter the PDMS was peeled off the Si/SU-8 mould and the edges were trimmed with a scalpel. This two-tier layer of PDMS may then be bonded to a quartz back-plate by exposing both interfacing surfaces to 30 seconds of 40 W oxygen plasma then bringing the surfaces in contact and baking at 120 °C for 10 minutes.

Any resist illuminated by UV radiation is susceptible to curing, therefore an aperture is beneficial. To create an aperture the quartz back-plate may be selectively coated in metal using traditional photolithography techniques. A 50 nm thick film of aluminium following the perimeter of the mesa was found to be effective. In addition, opaque tape may be used to secure the PDMS mesa onto the quartz which consequently acts as a second aperture. Figure S3 displays photos of a stamp before and after the application of opaque tape.


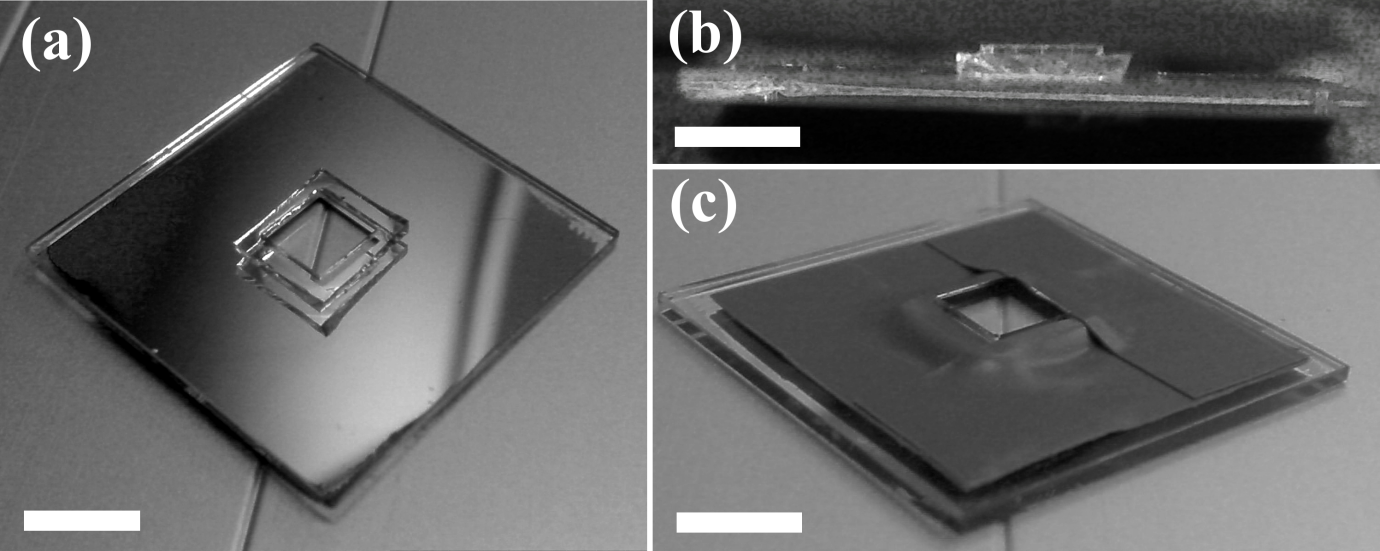


Figure S3. (a) – Photograph of a 25 mm square quartz mount with 50 nm thick evaporated Al creating a 5 mm square aperture for a 5 mm square PDMS mesa, scale bar = 7 mm. (b) - cross section view of the stamp from image ‘(a)’, scale bar = 5 mm. (c) – stamp from image ‘(a)’ with opaque tape, scale bar = 7 mm.

S3. Si etch parameters

To transfer patterns from Delo-Katiobond to Si the resist was first ashed with a 100 watt oxygen plasma for 1 minutes to remove any residual layer. The Si etch chemistry utilised was C_4_F_8_ /SF_6_ = 90/30 sccm, 600 W , 9.8 mTorr, 20 °C for 30 seconds in an Inductively Coupled Plasma (ICP) etch tool from Surface Technology Systems providing a Si etch rate of 150 nm/min. An etch selectivity of 4.87 was achieved against the Delo Resist. The etch profile was clean with a sidewall angle of 89^O^, as shown in the manuscript. It can also be observed from this figure that as well as having a strong Si etch selectivity, the imprints produced with this stepper are of high aspect ratio, typically 2.65:1, so deep Si etching is possible with this resist.

S4. Stepper accuracy

In this adaption of the CNC machine the accuracy of the X and Y position is of most relevance between a minimal movement length (equal to the smallest side of a mesa (5 mm)) and a maximum movement length (equal to the width of substrate). Table S1 exhibits the absolute and relative error (as measured using an FEI Novo NanoSEM 630) for one and eighteen 5 mm movements in both the X and Y direction at different operating speeds respectively. To analyse the stepped pattern a scanning electron microscope with calibrated optics and stage was used to first identify a feature on a known imprint, then the stage was repositioned so that the crosshairs of the microscope were situated upon the same feature on a subsequent imprint. By recording the X and Y position of the microscope stage, the change in lateral distance between imprints may be deduced and the error between the actual and expected value calculated. Table S1 shows that for all head speeds and displacements tested the relative error did not exceed 0.5% (which corresponds to 23 µm for a single 5 mm movement). Furthermore the absolute error is shown to be independent of head speed.

| Direction | Speed  (mm/sec) | No# of 5 mm Steps | Absolute Error (mm) | Relative Error (%) |
| --- | --- | --- | --- | --- |
| X | 3 | 1 | 0.012 | 0.240 |
| X | 3 | 18 | 0.012 | 0.013 |
| X | 14 | 1 | 0.018 | 0.365 |
| X | 14 | 18 | 0.027 | 0.030 |
| X | 25 | 1 | 0.016 | 0.323 |
| X | 25 | 18 | 0.002 | 0.002 |
| Y | 3 | 1 | 0.017 | 0.340 |
| Y | 3 | 18 | 0.023 | 0.031 |
| Y | 14 | 1 | 0.019 | 0.395 |
| Y | 14 | 18 | 0.037 | 0.041 |
| Y | 25 | 1 | 0.023 | 0.459 |
| Y | 25 | 18 | 0.023 | 0.025 |

Table S1. Stepping accuracy for different head speeds.

S5. Example G-code for performing a step-and-repeat cycle

%

O1 ( program name = 1)

G21 ( program in mm)

G40 G54 G90 (g40 Tool radius compensation off)

(g54 relates program zero directly to machine zero)

(G90 = absolute positioning, G91 = relative.)

G00 X10 Y20 Z0 (goto this absolute position)

G50 S10 (Set maximum spindle POWER TO 10)

G91 (switch to relative positioning)

z160 ( **** set z imprint level in mm (ie 160 mm) ***)

S10 M3 (spindal (LED) on FULL POWER)

G4 H30.0 (dwell for 30 seconds (software maximum) REPEAT step for prolonged curing)

M5 (SPINDAL (LED) OFF)

z180 (*** set z move position in mm (ie 180 mm)***)

X10 F150 (move one mesa in x (ie 10 mm square mesa))

z160 (**** set z imprint level in mm ***)

S10 M3 (spindal (LED) on FULL POWER)

G4 H30.0 (dwell for 30 seconds)

M5 (SPINDAL OFF)

z180 (*** set z move position in mm ***)

x-10 Y10 F150 (move back to the initial x position and move one mesa in y for exemplary raster scan)

G28 (return home)

M00 (stop and wait for manual continue)

%

S6. S1805 profile analysis


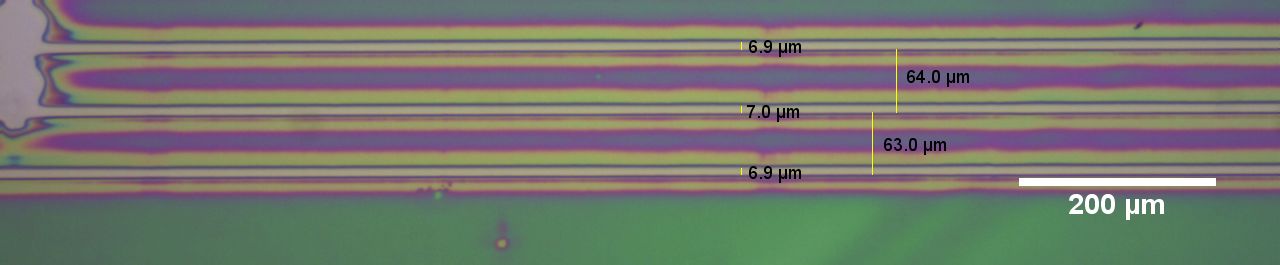

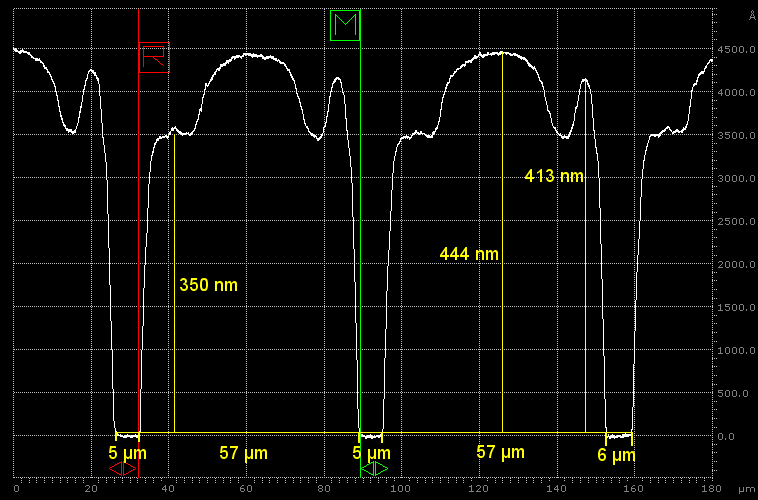


Figure S4. (top) Optical micrograph of S1805 resist featuring a double exposure with colour variation in the resist. (bottom) Depth profile of the same resist generated by a Veeco Dektak tool.

| S1805 Resist colour | Approximate corresponding thickness in nm |
| --- | --- |
| Green | 500-460 |
| Purple | 450-410 |
| Yellow | 370-320 |
| White | 0 |

**Table S2. Thickness range deduced by profile analysis for prominent colours visible in the images of S1805 on Si shown in Figure 3 of the manuscript.**

S7. UV power plots


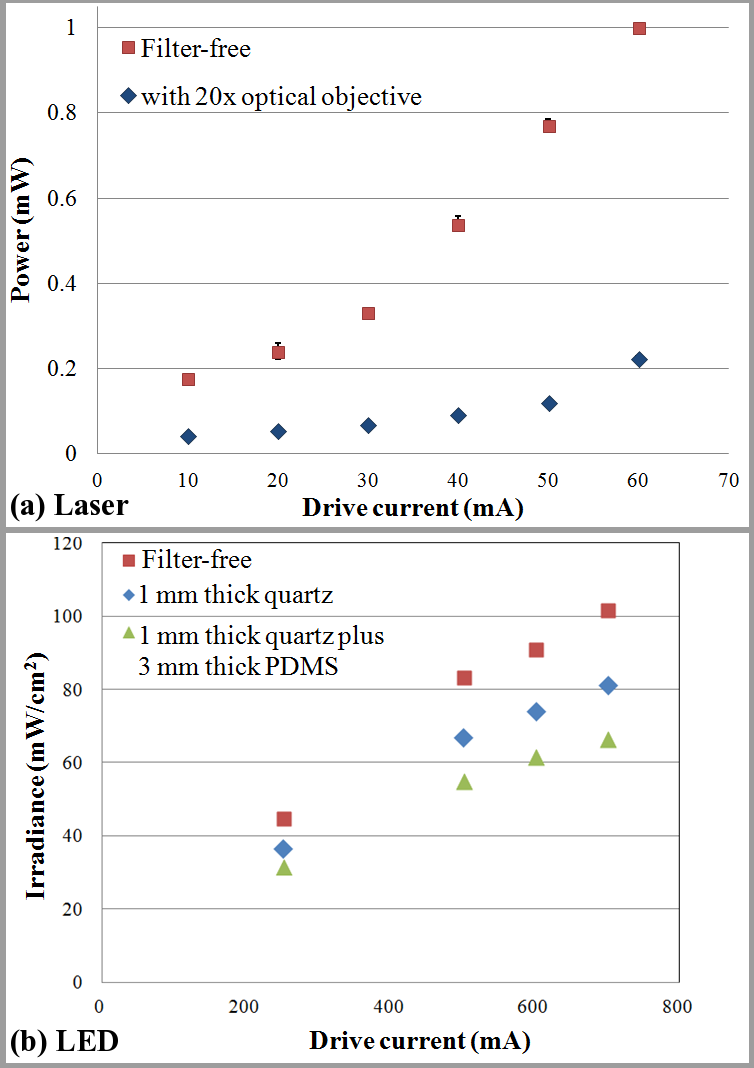


Figure S5. Plots of (a) laser spot power and (b) LED output irradiance against drive current in the presence and absence of appropriate filters. A minimum of three measurements were performed at each specified current level for both UV sources but the standard deviation is so small for each reading that the error bars are not visible in the graph.
